# Response: Commentary: The Developmental Trajectory of the Operational Momentum Effect

**DOI:** 10.3389/fpsyg.2019.00160

**Published:** 2019-02-06

**Authors:** Daniele Didino, Pedro Pinheiro-Chagas, Guilherme Wood, André Knops

**Affiliations:** ^1^Department of Psychology, Faculty of Life Sciences, Humboldt-Universität zu Berlin, Berlin, Germany; ^2^Laboratory of Behavioral and Cognitive Neuroscience, Stanford Human Intracranial Cognitive Electrophysiology Program, Department of Neurology and Neurological Sciences, Stanford University, Stanford, CA, United States; ^3^Department of Psychology, University of Graz, Graz, Austria; ^4^BioTechMed-Graz, University of Graz, Graz, Austria; ^5^CNRS UMR 8240, Laboratory for the Psychology of Child Development and Education, Paris, France; ^6^University Paris Descartes, Sorbonne Paris Cité, Paris, France

**Keywords:** operational momentum, mental arithmetic, numerical cognition, spatial biases, approximate mental calculation, number-to-line mapping tasks

Fischer et al. (2018) (henceforth: FM&S) raised theoretical and methodological criticisms against our study (Pinheiro-Chagas et al., 2018) on the development of the operational momentum effect (OM). Here, we will refute their criticisms and argue for a more precise definition of the OM as the operation-induced misestimation of arithmetic problem outcomes.

First, FM&S advocate the idea that zero-problems (e.g., 6+0) would be ideally suited to reveal OM. FM&S ask “*how does* [the attentional shift] *account explain larger OM with zero problems?*” In Pinhas and Fischer (2008) task, zero problems only required to map a number (the first operand) onto a labeled line, since these problems are solved by means of rules (i.e., N+0 = N, N−0 = N) rather than mental calculation (Butterworth et al., 2001; Campbell and Metcalfe, 2007). Therefore, FM&S's question is not valid because its premise (i.e., zero-problems produce OM) is not valid. Since zero and non-zero problems do not invoke the same strategies, merging their respective biases will not be helpful in elucidating the underlying mechanisms. The attentional shift account aims to describe the operation-specific outcome misestimations caused by mentally combining (at least) two numerosities. FM&S further argue that a stronger bias for zero problems compared to non-zero problems (Pinhas and Fischer, 2008; Shaki et al., 2018) invalidates the compression account of the OM “*because the logarithm of zero is not defined*.” This argumentation is flawed because FM&S mix up logarithm as a mathematical function (not defined for zero, indeed) with logarithm as a model (coding scheme) to describe the compressed internal scale of the representation of magnitudes (Nieder and Miller, 2003; Harvey et al., 2013). In the latter case, the logarithmic function is used as mathematical approximation of the relation between external physical magnitude and its internal representation. However, it makes no sense to assume that cortical circuits actually compute the faithful “mathematical log transformation” of a given sensory information. The intensity of external physical stimuli is internally represented via non-linear spatio-temporal neural codes (e.g., rate code, population code). Basing their criticism on the restriction of the mathematical definition of the logarithm to positive real numbers, FM&S conflate the mathematical definition with the neural and cognitive representation of magnitudes. Moreover, even assuming that the cognitive system would actually be bound to this particular *mathematical* formulation of the relation between physical stimulus magnitude and sensation, another framework has been put forward that does define a mathematical solution of zero magnitudes. Stevens's power function (with positive real exponents smaller than 1) can provide identical predictions and is defined for zero. In sum, the fact that “*the logarithm of zero is not defined”* does not invalidate the compression account nor seems the use of zero problems ideal for investigating OM.

Second, we argued that the attentional shift account and the heuristic account provide equivalent predictions. Fischer and colleagues criticize this by stating that it is in conflict with results from McCrink and Hubbard and cite: “…*the use of heuristics is generally increased when attention is decreased*” (McCrink and Hubbard, 2017, p. 240). Our interpretation of McCrink and Hubbard's manuscript was based on the idea that these two accounts “*are actually so deeply intertwined that they are indistinguishable*” (p. 240) and on the fact that McCrink and Hubbard's findings “*can be best described with a heuristics-via-spatial-shifts account*” (p. 241).

Third, FM&S criticize that the downward (upward) movement of addends (subtrahends) would be inconsistent with “*the vertical MNL*” and ask “*why* […] *operations along a horizontal MNL* [were] *primed with vertical movements?*” We argue that these movements actually mimic our daily experience: adding objects from the top into a box (downward movement) and subtracting them from inside a box to the top (upward movements). Any effect of this supposed inconsistency between physical vertical movements of the operands and attentional movement on the MNL should have weakened, eliminated or even reversed the OM. Yet, we did not observe such interference. They also reasoned that the center-to-top movement of the subtrahends “*removed attention from the place of mentally simulating the outcome, thus impeding subtraction*.” First, this conclusion is inconsistent with findings from previous studies (McCrink et al., 2007; McCrink and Hubbard, 2017), where OM was observed despite subtrahends moving to the right (i.e., inconsistently with the *horizontal MNL*). Second, FM&S conflate mental simulation of addition and subtraction with attentional focus in external space. After all, the outcomes are estimated in the participants' minds—not in external space where no numerical information is present at that point in time.

Finally, the idea that in our previous studies “*the normal ingredients of OM are dis-ordered or diluted*” originates from the divergent definition of the OM. In line with the original definition by McCrink et al. (2007), we propose that OM emerges during mental calculation, rather than rule application or arithmetic fact retrieval, and refers to the numerical deviation in estimated outcomes of arithmetic operations (e.g., addition vs. subtraction), rather than biases resulting from mapping outcomes to a non-numerical dimension. In number-to-line mapping tasks, participants locate addition and subtraction outcomes on a labeled line (Pinhas and Fischer, 2008) or modify the length of a line proportionally to addition and subtraction outcomes (Shaki et al., 2015, 2018). These paradigms do not measure outcome deviations, but rather they require an additional transformation process where the outcome is converted into another physical dimension (number to position or length). Both tasks can be subject to strategical (e.g., use of reference points; Barth and Paladino, 2011; Slusser et al., 2013; Sasanguie et al., 2016; but see Opfer et al., 2016) or procedural biases (e.g., perceptual hysteresis). Therefore, any observed biases may arise from the additional transformation process rather than the calculation process itself. Results from procedures that analyse only the final location on a labeled line (Pinhas and Fischer, 2008) or the length of a segment (Shaki et al., 2015, 2018) must be interpreted cautiously because they are not measuring OM but biases that may well take place after the calculation process and have their origin in the transformation algorithm.

## Author Contributions

DD and AK wrote the manuscript. PP-C and GW provided critical revision. All authors approved the final version of the manuscript for submission.

### Conflict of Interest Statement

The authors declare that the research was conducted in the absence of any commercial or financial relationships that could be construed as a potential conflict of interest.
